# Croup

**Published:** 1889-05

**Authors:** L. P. Foster

**Affiliations:** Minneapolis, Minn.


### CROUP.

Mr. Editor:—I like your criticisms on the article by P. P. Wells, in January number, giving Bœnninghausen's treatment of croup. It is not homœopathic nor scientific. I also take exceptions to the cases cited as membranous. My experience has taught me that true membranous croup seldom, if ever, begins before midnight, and comes on so deceptively that the friends do not get alarmed until the membrane is well formed and has been two or three days growing. Usually, true membranous croup begins about four A. M., and the first night the child only coughs a few times and feels as well the next day as if the croup or cough had never occurred. The second night at four A. M. the cough is repeated, with aggravations, and the next day the child seems quite well. The third night and day the case becomes more alarming and the physician is called, to find either a Kali-bi., Brom., or Iod. case—seldom, if ever, a Hepar or Spongia case. There is no necessity for a mistake for the physician to make if he will observe his case, slowly, and be governed by the following brief points in selecting his remedy:

Cough before midnight remedies are Acon., Spongia, and Hepar.

*Aconite.* Dry, hot, feverish, thirsty, with labored breathing. The child awakens from sleep coughing; he turns over, goes to sleep, and is awakened with cough again.

Hepar. Hoarse, deep cough; child *chokes* when coughing with *rattling* of mucus in chest.

*Spongia.* Dry, hollow cough with wheezing or asthmatic breathing; *no rattling* as in Hep. The coughs sounds like a saw sawing a board.

Remedies after midnight, Sambucus and Kali-bi.

*Sambucus.* Wheezing, hoarse, suffocating cough; *child sits up in bed to breathe and cough.*

Kali-bi. Cough with metallic sound; wheezing or rattling in larynx; expectoration of a tough, stringy, viscid mucus.

Bromium. Great prostration; child white, delicate skin, blue eyes; hoarse, loose, *rattling cough.*

*Iodium. No prostration;* child with dark skin and eyes; a *wheezing, sawing respiration,* and the child grasps the throat when coughing.

In Bromium there is prostration and rattling.

In Iodium there is no prostration and no rattling, but wheezing and a difference in color of skin and eyes.

*Phosphorus.* Dry cough, with pain in larynx when speaking; the child says it hurts him to talk.

Other remedies may be indicated, but these are the more common ones called for, and I must say, since using the high potencies, my success far surpasses any treatment in former years with the low.

L. P. Foster, M. D.

Minneapolis, Minn.

